# Effects of the Novel LaPLa-Enriched Medium- and Long-Chain Triacylglycerols on Body Weight, Glycolipid Metabolism, and Gut Microbiota Composition in High Fat Diet-Fed C57BL/6J Mice

**DOI:** 10.3390/molecules28020722

**Published:** 2023-01-11

**Authors:** Jinyuan Shi, Qianqian Wang, Chuang Li, Mengyu Yang, Muhammad Hussain, Junhui Zhang, Fengqin Feng, Hao Zhong

**Affiliations:** 1 College of Food Science and Technology, Zhejiang University of Technology, Hangzhou 310014, China; 2 College of Biosystems Engineering and Food Science, Zhejiang University, Hangzhou 310058, China

**Keywords:** MLCT, lipid metabolism, glucose homeostasis, gut microbiota, inflammation

## Abstract

The roles of medium- and long-chain triacylglycerols (MLCT) on health benefits under high fat diet (HFD) conditions remain in dispute. This study was conducted to investigate the effects of novel LaPLa-rich MLCT on the glycolipid metabolism and gut microbiota in HFD-fed mice when pork fat is half replaced with MLCT and palm stearin (PS). The results showed that although MLCT could increase the body weight in the mouse model, it can improve the energy utilization, regulate the glucose and lipid metabolism, and inhibit the occurrence of inflammation. Furthermore, 16S rRNA gene sequencing of gut microbiota indicated that PS and MLCT affected the overall structure of the gut microbiota to a varying extent and specifically changed the abundance of some operational taxonomic units (OTUs). Moreover, several OTUs belonging to the genera *Dorea, Streptococcus*, and *g_Eryipelotrichaceae* had a high correlation with obesity and obesity-related metabolic disorders of the host. Therefore, it can be seen that this new MLCT has different properties and functions from the previous traditional MLCT, and it can better combine the advantages of MLCT, lauric acid, and sn-2 palmitate, as well as the advantages of health function and metabolism. In summary, this study explored the effects of LaPLa-enriched lipids on glycolipid metabolism in mice, providing theoretical support for future studies on the efficacy of different types of conjugated lipids, intending to apply them to industrial production and subsequent development of related products.

## 1. Introduction

Medium- and long-chain triacylglycerols (MLCT), which always involve essential fatty acids, are structural lipids containing both medium chain triacylglycerols (MCT) and long chain triacylglycerols (LCT) in triglyceride molecules [1]. The metabolism of triglycerides is closely related to the composition and location distribution of fatty acids, which determine the structure of triglycerides [2]. MCT do not contain essential fatty acids, while MLCT and LCT could contain essential fatty acids, depending on the types of fatty acids attached to them. LCT differed greatly from MCT and short-chain fatty acid triglycerides (SCT) in human metabolism [3]. LCT, which has a relatively slow metabolic rate, is highly dependent on the carnitine transport system. While the metabolic rate of SCT and MCT is relatively fast, which can be absorbed directly through emulsification [4]. MLCT overcomes the disadvantage of LCT in metabolism, and can be hydrolyzed in the absence of pancreatic lipase [5]. At the same time, MLCT can be absorbed directly by entering the portal vein and absorbed in the lymphatic system into the blood port through the formation of chylomicron, independent of the transporter [6].

With the improvement of social living standards, and the changes in diet and lifestyle, the incidence of obesity has increased, and the related diseases caused by obesity have been paid more attention. In 2005, the number of obese and overweight people worldwide reached 1.3 billion, and the figure is expected to increase to 2 billion by 2030 [7]. Importantly, in terms of physiological function, MLCT can effectively prevent obesity, reduce the lipid and cholesterol content of obese patients, and prevent hyperglycemia and diabetes to a certain extent [8]. Compared with traditional fats and oils, MLCT, which contains essential fatty acids, will not cause fat accumulation or ketone body poisoning while providing energy for the body [9]. In addition to being thought to have a role in managing obesity, MLCT can also regulate diabetes by improving insulin resistance [10,11]. Recent evidence suggested that 3-week use of MLCT reduced glucose levels and increased insulin secretion to delay the progression of hyperglycemia in diabetic OB/OB mice [12]. MLCT can effectively reduce the level of anti-inflammatory factors caused by hyperlipidemia, alleviate the excessive accumulation and inflammatory response of adipose tissue in obese rats to a certain extent, restrain atherosclerosis, and improve liver lipid metabolism by regulating the expression of lipid metabolite-related enzymes [13].

The research and development of MLCT was initially aimed at people with lipid metabolism disorders and assisted in the improvement of obesity caused by excessive intake of fat, as well as various complications [14]. Along with the in-depth study, the application of MLCT is increasingly concentrated in glycolipid regulation, especially for people with fast-paced life, high fat intake, and lack of exercise [15]. In previous studies, MLCT research and development mostly use caprylic acid as the raw material of medium-chain fatty acids, which have shown good effects in animal and human trials, such as lowering blood lipids, lowering cholesterol, and raising high-density lipoprotein [16]. Lauric acid (La), containing 12 carbon atoms, is an ideal medium-chain fatty acid (MCFA) to incorporate into LCTs to synthesize MLCTs, as it exhibits antibacterial activity, anti-diabetes properties, and is anti-inflammatory [17]. On the other hand, studies revealed that sn-2 palmitate could increase the growth of the intestinal probiotics, improve bone density, and promote brain development [17]. Thus, LaPLa, a new type of MLCT, which combines the metabolic advantages of MLM-type MLCT with the efficacy of lauric acid and sn-2 palmitate, is a new structured lipid that does not contain any essential fatty acid, needs to be investigated in further study [18]. As a new type of MLCT, it is worthy studying whether LaPLa-enriched MLCT has similar functions to traditional MLCT, especially regarding lipid metabolism [19].

The gut microbiota is now considered to be a key factor in metabolic health. Different dietary lipid compositions have different effects on gut microbiota structure, and an unbalanced dietary lipid structure may lead to intestinal dysbiosis, which may lead to intestinal inflammatory diseases [20]. High-fat diets or diets rich in saturated fat have adverse effects on the gut microbiota, which are manifested as decreased richness and diversity of the gut microbiota and an increased Firmicutes/Bacteroidetes ratio, which may lead to increased levels of intestinal pro-inflammatory cytokines and increased intestinal mucosal permeability [21]. Recent evidence suggests that MLCT can affect the composition of intestinal flora in mice. The diet supplemented with MLCT significantly reduced the ratio of Firmicutes to Bacteroides, and down-regulated the relative abundance of Proteobacteria, thus regulating lipid metabolism in mice [22]. Furthermore, MLCT with different MCFA contents did not affect the species diversity of intestinal microflora, which reached 97% homology [23].

In this study, 50% of the lard in the high-fat diet was replaced by MLCT as the experimental group, and the high-fat diet group (HFD), basic diet group (NCD), and palm stearin group (PS) were set as the control group. It is consistent with the replacement ratio of MLCT feed lard. In this way, it is a preliminary exploration of the function of a novel MLCT, to study the effect on the lipid metabolism of mice, to provide a theoretical basis for evaluating the function of a new type of lauric acid-rich MLCT, and to lay a foundation for the subsequent development of similar products.

## 2. Results

### 2.1. Body Weight Gain and Energy Intake

**Figure 1:**
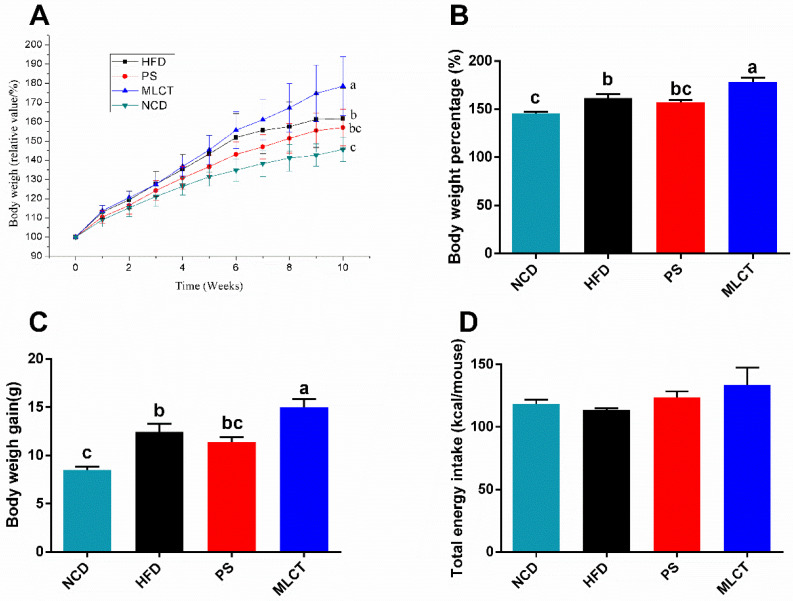
Body weight gain and energy intake. (**A**) Body weight of mice over 10 weeks. (**B**) Body weight gain percentage. (**C**) Body weight gain. (**D**) Total energy intake. In (**B**,**C**), means with the different lowercased letters differ significantly (*p* < 0.05) according to one-way ANOVA analysis followed by Tukey’s tests.

As shown in Figure 1, the average body weight percentage (BWP) of mice fed with different feeds varied differently. As expected, compared to the NCD group, the body weight gain and total body weight of mice in the HFD group increased significantly at the end of the experiment. Interestingly, following the feeding cycle increase, the MLCT group exhibited a rapid weight gain in comparison with the HFD and PS groups. There was no significant difference between the PS and HFD groups. Figure 1D indicated that there was no significant difference in the total energy intake among the four groups. However, compared to the other three groups, a slight increase in energy intake could be observed in the MLCT group (133.7 kcal).

### 2.2. Organ Indexes and Morphological Changes

**Figure 2:**
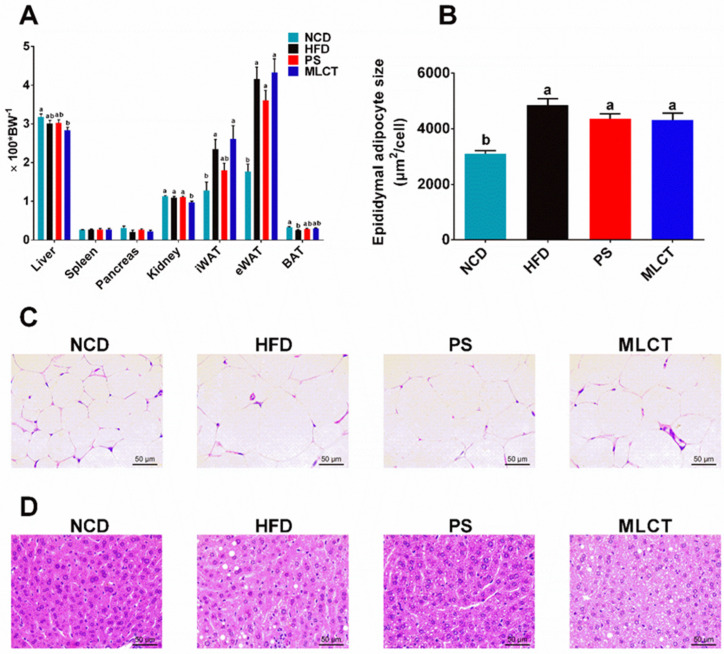
Organ indexes and morphological changes. (**A**) The weight percentage of organs. (**B**) Adipocyte size of eWAT (*n* = 6). (**C**) H&E staining of eWAT. (**D**) H&E staining of liver tissues. In (**B**), means with the different lowercased letters differ significantly (*p* < 0.05) according to one-way ANOVA analysis followed by Tukey’s tests.

After 10 weeks of feeding, there was a significant increase in the percentage weight of iWAT and eWAT in the HFD and MLCT groups (Figure 2A). In comparison, supplementation with PS slightly decreased the iWAT and eWAT weight. These results were in accordance with the body weight gain results. In terms of spleen and pancreas index, all four groups showed no significant differences. A significant reduction of the weight of the liver and kidney index in the MLCT group was found compared to the other three groups. The BAT index in the HFD group was significantly decreased compared to the NCD group. The adipocyte size of eWAT in the HFD group was noticeably increased compared to the NCD group, whereas PS and MLCT treatments reduced the increase induced by the HFD condition (Figure 2B,C). The histopathological changes in the liver tissue are shown in Figure 2D, and the number of white lipid droplets in the liver biopsies of the HFD group was significantly larger than that of the NCD group, suggesting adverse hepatic lipid accumulation. In comparison with HFD-fed mice, a significant decrease in the fat accumulation of hepatocytes was observed after PS and MLCT supplementation.

### 2.3. Serum Lipids

**Figure 3:**
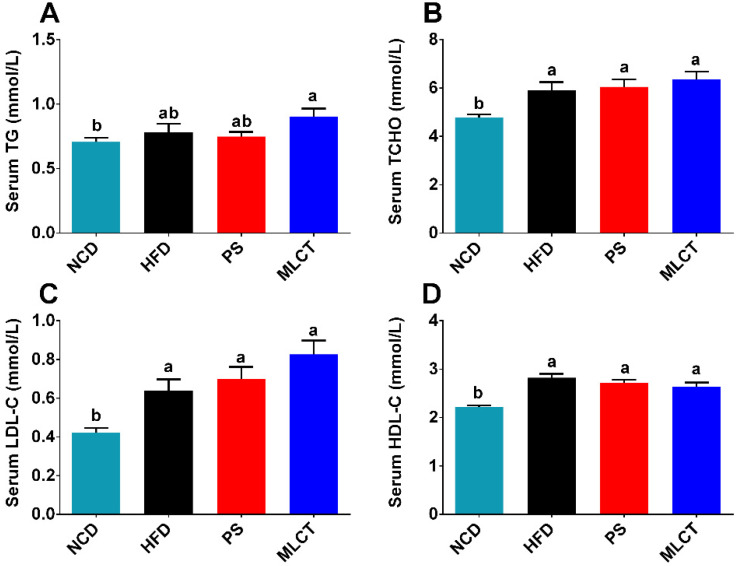
Effects of MLCT on serum lipid profiles. (**A**) TG. (**B**) TCHO. (**C**) LDL-C. (**D**) HDL-C. Data were shown as mean ± SE (*n* = 8). In (**A**–**D**), means with the different lowercased letters differ significantly (*p* < 0.05) according to one-way ANOVA analysis followed by Tukey’s tests.

Generally, the changes in serum lipids are closely related to the body weight. The level of lipid metabolism is reflected by the four serum lipids (TG, TC, LDL-C, and HDL-C) [24]. Figure 3A indicated that only MLCT significantly increased the TG level in comparison with the NCD group. In addition, no significant differences among the HFD, PS, and MLCT groups were found. As shown in Figure 3B–D, compared to the NCD group, the three groups (HFD, MLCT, and PS) exhibited significant increases in three parameters (TCHO, LDL-C, and HDL-C), whereas no differences were observed among these groups. All these results suggest that dietary intake of MLCT has little influence on regulating serum lipid metabolism.

### 2.4. Glucose Homeostasis

**Figure 4:**
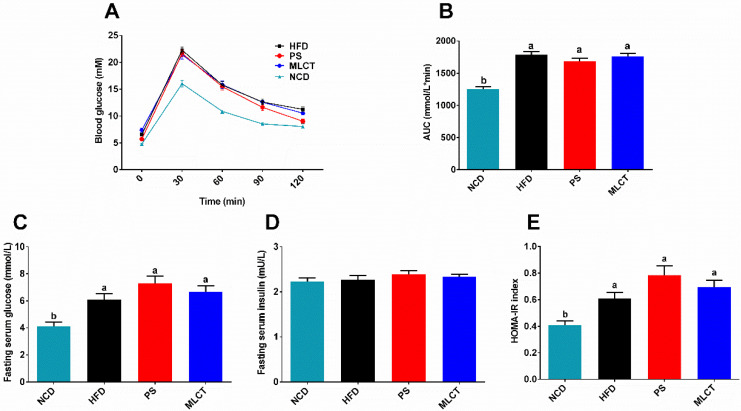
Effects of MLCT on glucose homeostasis. (**A**) Glucose tolerance test (GTT). (**B**) Area under curve (AUC) from GTT. (**C**) Fasting serum glucose. (**D**) Fasting serum insulin. (**E**) HOMA-IR. Data were shown as mean ± SE (*n* = 8). In (**B**,**C**,**E**), means with the different lowercased letters differ significantly (*p* < 0.05) according to one-way ANOVA analysis followed by Tukey’s tests.

Obesity is always accompanied with glucose metabolism disorders and insulin resistance. In the case of GTT, Figure 4A,B indicated that HFD triggered insulin resistance and less glucose tolerance. After 60 min, the PS group has a faster drop in blood glucose than that of the MLCT and HFD groups. After 90 min, the blood glucose of the MLCT group decreased faster than in the HFD group. The area under the GTT curve (AUC) is presented in Figure 4B. Obviously, there is no significant difference among the three groups of HFD, MLCT, and PS, indicating that the MLCT group did not significantly decrease the glucose tolerance due to rapid weight gain, especially compared with the HFD group. Moreover, the results of the fasting blood glucose were shown in Figure 4C; three treatments (HFD, MLCT, and PS) significantly increased the serum glucose levels in comparison with the NCD group, and no significant differences were found among the three groups of HFD, MLCT, and PS. There is no significant difference in serum insulin levels among all groups (Figure 4D). Likewise, Figure 4E indicated that there was no difference in insulin resistance performance among three treatments. These data suggest that MLCT has little impact in regulating serum glucose metabolism.

### 2.5. The Effect of MLCT on Hormones, Inflammatory Response, and Endotoxin in Mice

**Figure 5:**
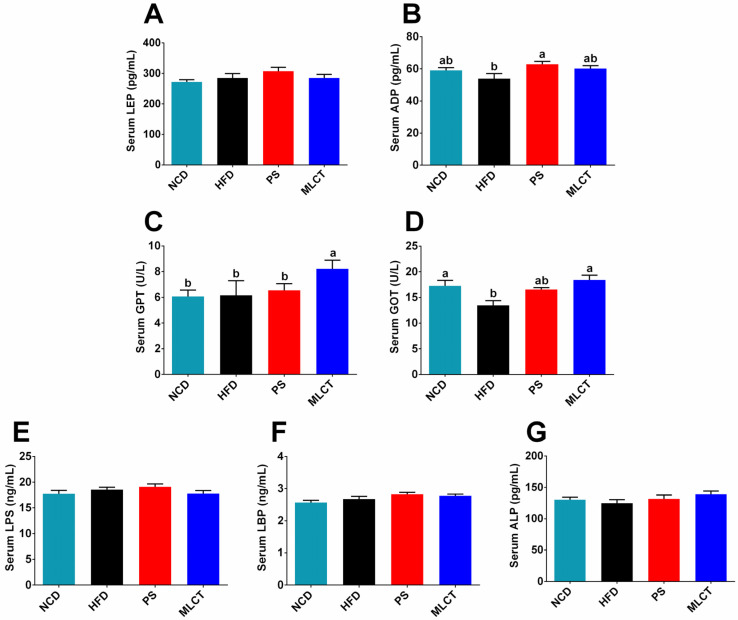
Effects of MLCT on serum hormones and cytokines. (**A**) LEP. (**B**) ADP. (**C**) GPT. (**D**) GOT. (**E**) LPS. (**F**) LBP. (**G**) ALP. Data were shown as mean ± SE (*n* = 8). In (**B**–**D**), means with the different lowercased letters differ significantly (*p* < 0.05) according to one-way ANOVA analysis followed by Tukey’s tests.

Leptin and adiponectin are two important lipid metabolism regulators. As identified in Figure 5A, no significant difference in leptin among the four groups was observed, revealing that the rapid weight gain of MLCT did not affect leptin secretion. Figure 5B indicated that adiponectin of PS group showed a significant increase compared to the HFD group, which may be related to the weight loss trend in PS group.

The glutamic-pyruvate transaminase (GPT) and glutamic-oxalacetic transaminase (GOT) activities are positively related to the inflammatory status, especially the occurrence of liver disease. Figure 5C revealed that the MLCT group had a significantly higher GPT level than that of other three groups. In terms of GOT, HFD group had the lowest level, and proposed a significant difference from MLCT group and NCD group.

Lipopolysaccharide (LPS) is an endotoxin that causes inflammation. Lipopolysaccharide binding protein (LBP) is a reactive protein whose content increases in response to LPS. In addition, inflammation is always accompanied by high levels of serum alkaline phosphatase (ALP) [13]. The results of LPS, LBP, and ALP are presented in Figure 5E–G. No significant differences were found among four groups in serum LPS, LEP, and ALP, suggesting that the inflammatory response caused by rapid weight gain in MLCT was not significantly changed.

### 2.6. Organ Indexes and Morphological Changes

**Figure 6:**
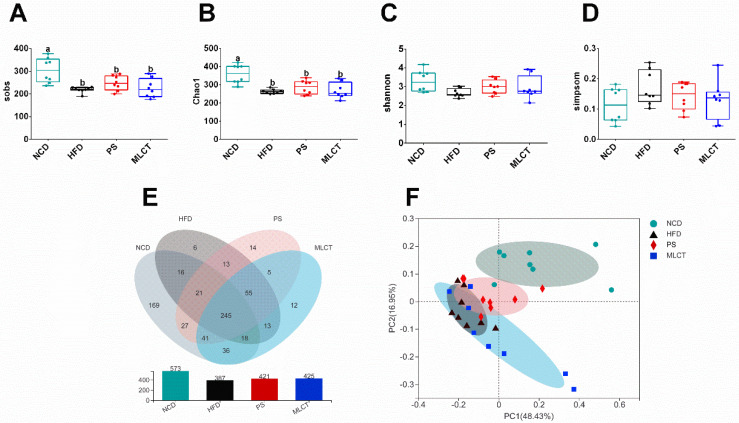
Effects of MLCT on diversity of the gut microbiota. (**A**) Sobs. (**B**) Chao1. (**C**) Shannon. (**D**) Simpson. (**E**) OTUs Venn Diagram. (**F**) PCoA plot (weighted UniFrac distances). Data were shown as mean ± SE (*n* = 8). In (**A**–**D**), means with the different lowercased letters differ significantly (*p* < 0.05) according to one-way ANOVA analysis followed by Tukey’s tests.

We investigated whether MLCT can exert a healthy effect on weight gaining by affecting the composition of the gut microbiota. The Sobs and Chao1 indices were decreased in HFD, PS, and MLCT compared to the NCD group (Figure 6A,B), while there were no differences between all groups in Shannon and Simpson indices (Figure 6C,D). Figure 6E showed that the PS group and the MLCT group had 421 and 425 OTUs, respectively, which was more than the HFD group (387). The PCoA results indicated that there was a certain distance between the NCD group and the HFD-fed groups, and the PS group was closer to the NCD group compared to the MLCT and HFD groups (Figure 6F).

**Figure 7:**
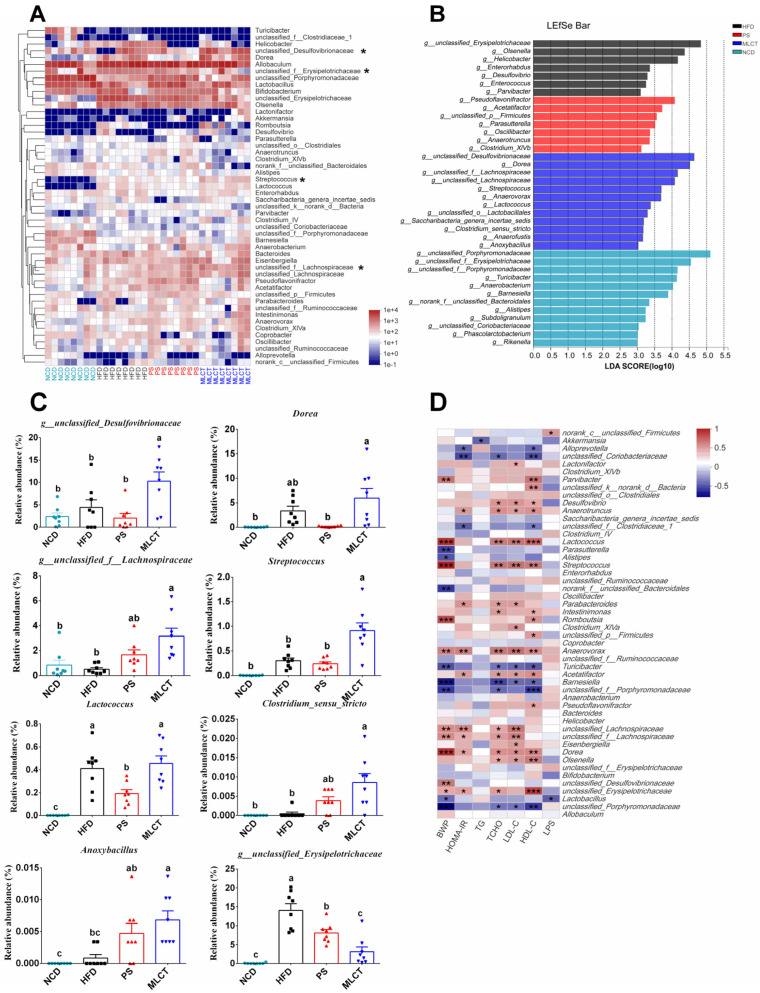
Effects of MLCT on the gut bacteria composition. (**A**) Heatmap analysis on genus level (the relative abundance of OTUs > 0.25%); (**B**) The LEfSe analysis (LDA > 3.0). (**C**) The relative abundance of *g_unclassified*_*f_Desulfovibrionaceae*, *Dorea*, *g_unclassified_f_Lachnospiraceae*, *Streptococcus*, *Lactococcus*, *Clostridium_sensu_stricto*, *Anoxybacillus*, and *g_unclassified_Erysipelotrichaceae*. (**D**) Correlations between intestinal microflora and biochemical indicators. In (**A**), *p* < 0.05 marked with “*” compared with HFD group. In (**D**), *p* < 0.001 marked with “***”, *p* < 0.01 marked with “**”, *p* < 0.05 marked with “*”. In (**C**), means with the different lowercased letters differ significantly (*p* < 0.05) according to one-way ANOVA analysis followed by Tukey’s tests.

After classification, the intestinal microbiota in the four groups mainly consisted of 50 genera (the relative abundance of OTUs > 0.01%, Figure 7A). The consumption of a HFD significantly increased the abundance of *g_unclassified_Erysipelotrichaceae*, *Olsenella*, *Dorea*, *Helicobacter*, *Acetatifactor*, *unclassified_p__Firmicutes*, *Anaerovorax*, *Romboutsia*, *Lactococcus,* and *Parvibacter*, and significantly decreased the relative abundance of *unclassified_Porphyromonadaceae*, *Turicibacter*, *Barnesiella*, *unclassified_Coriobacteriaceae*, *Alloprevotella,* and *norank_f__unclassified_Clostridiales* compared to the NCD group. To further examine the specific effects of half replacement of PS and MLCT on the gut microbiota in HFD-fed mice, an LEfSe analysis was used to find the distinct bacteria in each group (Figure 7B). Figure 7C showed the modifications of the abundance of the characterized genera of bacteria base on LEfSe report. The predominant genus in the NCD group was *unclassified_Porphyromonadaceae* and in the HFD-fed mice was *g_unclassified_Erysipelotrichaceae* (Figure 7B). The obese-type gut microbiota (*g_unclassified_Erysipelotrichaceae*) were significantly decreased after PS and MLCT intervention (Figure 7C). Compared to the HFD group, MLCT intervention significantly increased these genera (*g__unclassified_Desulfovibrionaceae*, *g__unclassified_f__Lachnospiraceae*, *Streptococcus*, *Clostridium_sensu_stricto,* and *Anoxybacillus*; Figure 7C). Finally, Spearman’s correlation analysis was applied to investigate the relationship between the gut microbiota and biochemical profiles (Figure 7D). Microbes such as *unclassified_Desulfovibrionaceae, g__unclassified_f__Lachnospiraceae,* and *Streptococcus* were found to be significantly enriched in the MLCT group, and were positively correlated with BWP and LDL-C. In contrast, *Turicibacter*, *Barnesiella,* and *unclassified_Porphyromonadaceae* had negative correlations with TCHO and BWP.

## 3. Discussion

The present study was carried out to evaluate the effect of MLCT on body weight, glycolipid metabolism, and gut microbiota composition for 10 weeks in half replaced dietary fat conditions. There was a trend that the body weight was found to be the highest in LaPLa-enriched MLCT fed mice (*p* < 0.05). Contradictory to our experiments, in terms of body weight gain for the mice subjects, most studies showed MLCT did not significantly affect the body weight gain. It was suggested that the effect of MLCT on body weight might be influenced by the dietary fat content or by energy sufficiency [25]. Consistent with our studies, some MLCT studies also showed no significant difference in terms of fat pad accumulation for the mice subjects. The results show that the animals fed with LaPLa-enriched MLCT has a significant weight gain, especially greater than the HFD group, although the fat pad accumulation was not statistically significant (*p* > 0.05; Figure 2). This trend is similar to monoglyceride laurate in livestock and poultry production [26]. One study found that glycerol monolaurate (GML) could significantly improve daily body weight gain and feed conversion rate in broiler subjects [27]. In addition, other studies reported that the addition of GML-enriched coconut oil into the diet of pregnant sows could significantly increase the fat content in milk and increased the birth weight of piglets by 10~12% [28]. This may be due to LaPLa and GML being metabolized as La, which could increase the energy storage, thus inducing the body weight gain [29]. Combined with energy intake in mice, the MLCT group consumed relatively more energy (*p* > 0.05), which determines that the energy intake and the energy conversion rate of the MLCT group is higher than other groups.

In general, as an important index for lipid metabolism, the liver organ index can reflect liver injury and lipid accumulation well, which is associated with body weight gain [30]. In this research, the liver organ index of the MLCT group was significantly decreased, which showed a reverse trend of body weight gain. However, the MLCT did not cause over fat accumulation in mice. This suggested that healthy obesity may have few effects on metabolic abnormalities. A high-fat diet could significantly increase the inguinal fat index, which is a manifestation of obesity. In contrast, brown fat is beneficial to the body, which can break down white fat and convert it into CO_2_, water, and energy [31]. The above results reflect that no significant difference was found in the inguinal fat index between HFD and MLCT groups. In addition, the MLCT group had a higher brown fat index than the HFD group, which may explain the healthy obesity of MLCT. It is reported that the decrease in brown fat index in HFD group was more adverse to lipid metabolism, and epididymal fat is frequently detected in obesity animal experiments, which can reflect the accumulation of fat in mice [32]. Moreover, the liver and epididymal fat were stained and observed, and it was found that the fat particles formed in the liver of the MLCT group were smaller, but there was no significant difference in the epididymal fat area between the HFD group and PS group. It seems to be consistent with other research, which found that MLCT significantly inhibited the size of adipocytes and the number of macrophages, and reduced the levels of tumor necrosis factor—α, monocyte chemotactic protein-1, and endotoxin [33].

On the one hand, the HOMA-IR and AUC of the GTT are two indicators to evaluate insulin sensitivity [34]. Obesity always leads to decreased insulin sensitivity, and reduced glucose tolerance in turn, which is the main cause of type 2 diabetes [35]. In this research, the glucose tolerance of the HFD group, PS group, and MLCT group was lower than that of the NCD group, which is consistent with the law of the influence of the diet on glucose tolerance caused by obesity. A previous study found that after 3 weeks of feeding the mice with long chain triacylglycerol (LCT) or MLCT, the blood glucose was negatively correlated with total abdominal fat, and was significantly lower in the MLCT group than that in the LCT group, which is attributed to the higher insulin concentration [12]. On the other hand, the weight gain of the MLCT group has less impact on glucose homeostasis while quickly replenishing energy. Hence, it could conceivably be hypothesized that the intake of MLCT may promote insulin secretion and stimulates glucose uptake in adipose tissue, leading to increased abdominal mass. Further research should be undertaken to investigate the effects of MLCT on abdominal fat in human diabetic patients and diabetic obese mice. In line with the increased body weight, MLCT induced a significant increase in TG, TC, and LDL-C compared with the NCD group. However, there was no significant difference in the blood lipids of the three groups of HFD, PS, and MLCT, indicating that healthy obesity will not disturb lipid metabolism. Eventually, this also agrees with previous observations, which showed that MLCT containing 13.0% (W/W) MCFA can effectively increase HDL-C levels and reduce LDL-C concentration [36].

Leptin (LEP) mainly achieves effective regulation of body weight by balancing energy and suppressing appetite. Adiponectin (ADP) plays a protective role in diabetic nephropathy (DN) through the adiponectin receptor, mediates fatty acid metabolism, protects and compensates for further kidney damage, and prevents the development of clinical nephropathy [37]. The results indicate that MLCT had no effect on leptin levels in mice. Compared with the HFD group, the MLCT group showed an increasing trend of ADP, which suggested that MLCT prompts the body to produce more adiponectin in response to the adverse effects of obesity on glucose homeostasis and lipid metabolism. Inflammation always accompanies obesity and disorders of lipid metabolism. In this study, the GOT of the NCD group was significantly higher than that of the HFD group, which may be due to the malnutrition of the basic dietary diet, thus causing myositis [33]. In addition, there was no difference in LPS, LBP, and ALP among the four groups. Another study reported that MLCT could regulate inflammation [38]. However, to our best knowledge, no studies have shown the specific mechanisms through which MLCT regulates the inflammatory response of the body. Several questions remain unanswered at present [39].

In the following study, MLCT was found to have great influence on the overall structure of the gut microbiota, and the influence extent depended on the composition and dosage of MLCT. As expected, the ratio of Firmicutes to Bacteroidetes (F/B) in the HFD group was notably higher than the ratio in the NCD group (Figure S1). This is in good agreement with several studies in rodents and humans showing that a high F/B ratio is associated with a high fat diet, weight gain, and obesity [40]. Several studies have reported that MLCT could decrease the ratio of F/B [20]. However, this trend was not detected in the MLCT intervention groups in our study. It may be due to the different dosage and their different structures. Likewise, for the microbiota structure (α diversity and β diversity), there is no significant difference among the HFD-fed mice (HFD, PS, and MLCT).

On the basis of LEfSe analysis, the genera *g_Eryipelotrichaceae* and *Desulfovibrio*, which were the characteristic flora of the HFD group, were found to be positively correlated to the TCHO, suggesting that these bacteria may play a lipid-absorption role in the development of obesity [41]. The MLCT group is characterized by *Dorea* (Figure 7D), which was considered as an obesity biomarker, and positively associated with body weight [42]. Eryipelotrichaceae is a family associated with several health-damaging effects, and its presence was reported to increase the risk of inflammation. Interestingly, after intake of MLCT, the abundance of *g_Eryipelotrichaceae* was significantly decreased, indicating that MLCT exhibited the potential beneficial inhibition of against the inflammation-related bacteria *g_Eryipelotrichaceae*. Several bacterial species within the same family or genus were also found to respond differently to the HFD and MLCT intervention in this study. Indeed, the same species exist even within totally different impacts on host health. Thus, further studies are required to assess the effects of MLCT on obesity and the gut microbiota, and the isolation and culture of key bacteria strains are especially important. These findings referred that MLCT was a potent alternative in enriching the body weight gain-associated gut bacteria in HFD-fed mice. According to the chemical composition of MLCT is different from PS, and the similar trend as GML treatment, it is proposed that the changes in body weight, glycolipid metabolism, and the gut microbiota composition between the three treatment groups are mainly due to the variation of the fatty acid composition. Conclusively, MLCT replacement could possibly provide a robust modulation of the gut microbiota, which possesses a promising faster healthy weight-gain potential.

## 4. Materials and Methods

### 4.1. Materials and Chemicals

MLCT was prepared according to our previous study [17], the purity of LaPLa was 41.64 ± 0.51%, and the fatty acid composition of MLCT is shown in Table S1. Glucose, triglycerides (TG), total cholesterol (TCHO), high-density lipoprotein cholesterol (HDL-C), low-density lipoprotein cholesterol (LDL-C), alanine aminotransferase (GPT), and aspartate aminotransferase (GOT) kits were purchased from Nanjing Jiancheng Bioengineering Research Institute (Nanjing, China). Blood glucose test paper was obtained from Roche (Shanghai Roche Pharmaceutical Co., Ltd., Shanghai, China). Serum insulin, lipopolysaccharide (LPS), lipopolysaccharide binding protein (LBP), alkaline phosphatase (ALP), leptin (LEP), and adiponectin (ADP) ELISA kits were bought from Wuhan Genemei Biotechnology Co., Ltd. (Wuhan, China). Paraformaldehyde (4%) tissue fixative and adipose tissue fixative were purchased from Wuhan Google Biotechnology Co., Ltd. (Wuhan, China). Other related common reagents were supplied by Aladdin Reagent Co., Ltd., (Shanghai, China) or Sinopharm Chemical Reagent Co., Ltd. (Shanghai, China).

### 4.2. Animal Experiments Design

Male C57BL/6 mice, 3–5 weeks old, were raised in the SPF rodent breeding room of the Animal Experiment Center of Zhejiang University of Traditional Chinese Medicine. The temperature of the breeding environment was controlled at 22 ± 2 °C, and the light and dark cycle was alternated for 12 h. The relative humidity of the environment was 40~60%. After 1 week of adaptive feeding, the experimental mice were randomly divided into four experimental groups; each group had four cages with 12 mice (3/3/3/3) fed with different treatment diets. During the experiment, the mice could free access to food and water, feeding for 10 weeks. The specific experimental group design is as follows:

(1) High-fat diet group (HFD), fed with 206.8 g/kg lard in diet.

(2) The MLCT group, fed with 103.4 g/kg MLCT and 103.4 g/kg lard in diet.

(3) The palm stearin group (PS), fed with 103.4 g/kg PS and 103.4 g/kg lard in diet.

(4) The normal control diet group (NCD), fed with the basic diet.

Body weight and feed intake were regularly recorded every week. On the 9th week, the mice were fasted for 12 h and tested for glucose tolerance. After 10 weeks, the mice were fasted overnight, and fresh blood samples were collected from the orbit. Then cervical dislocation was sacrificed. The organs and tissues such as liver, epididymal white fat tissue (eWAT), inguinal white adipose tissue (iWAT), small intestine, and brown fat tissue (BAT) were dissected and collected. Except for intestinal tissues, the rest of tissues were quickly weighed and recorded. The liver was fixed in 4% paraformaldehyde tissue for a fixative solution, and some eWAT were fixed in fat fixation solution for follow-up morphological analysis. The remaining tissues were quick-frozen in liquid nitrogen and stored at −80 °C for subsequent analysis.

The animal experiment was approved by the Animal Ethics Committee of Zhejiang University of Traditional Chinese Medicine (Approval number: IACUC-20190408-14), and all experimental procedures were in accordance with the animal welfare regulations of Zhejiang University of Traditional Chinese Medicine.

### 4.3. Intraperitoneal Injection of Glucose Tolerance Test

The glucose tolerance test is namely the GTT test. On the 9th week of the feeding experiment, the fasting blood glucose of the mice was measured by taking blood from the tail vein after the mice were fasted for 12 h, which was recorded as 0 min blood glucose. Then 20% glucose solution was injected intraperitoneally at the dose of 2 g kg^−1^ body weight, and blood samples were taken for 30 min, 60 min, 90 min, and 120 min for blood glucose measurement. The glucose curve was drawn, and the area under the curve (AUC) was calculated according to the approximate trapezoidal method.

### 4.4. Determination of Serum Biochemical Indexes

The blood of mice was collected and stood at room temperature for 30 min, then centrifuged at 3000 rpm for 20 min. The upper serum was collected and placed in a sterilized 2.5 mL centrifuge tube every 50 μL. The samples were labeled and stored in a refrigerator at −80 °C for testing. The contents of glucose, TG, TCHO, HDL-C, LDL-C, GPT, and GOT in serum were determined according to the operation steps of Nanjing Jiancheng kit. While the contents of insulin, LPS, ALP, LBP, LEP, and ADP in the serum were determined according to the operating procedures of Genemei ELISA kit.

The insulin resistance index (*HOMA-IR*) is calculated according to the following formula:

$$HOMA\text{-}IR=\frac{Fasting\;serum\;glucose\;\left(mmol/L\right)\times Fasting\;serum\;insulin\;\left(\mu M/mL\right)}{22.5}$$

### 4.5. Morphological Analysis of Tissue Sections

Liver and eWAT were fixed in 4% paraformaldehyde tissue fixation solution and fat fixation solution for 24 h, then dehydration, paraffin embedding, sectioning (5 μm), and hematoxylin-eosin (H&E) staining were performed. After dehydration and mounting, pathological observation and analysis of tissue sections were performed under an optical microscope (CM1900, Leica, Germany). Five fields of view were randomly selected for each section for photographing. Finally, the area of adipocytes in epididymis was statistically analyzed by ImageJ software (version 6.0, Media Cybernetics, Rockville, MD, USA).

### 4.6. 16S rRNA Analysis of the Gut Microbiota

For gut microbiota analysis, the procedure was carried out according to our study [40]. Briefly, fecal total DNA (n = 8) was extracted using a QIAamp DNA Stool Mini Kit (QIAGEN, Venlo, Netherlands) following the manufacturer’s instructions. The V3-V4 hypervariable regions of the bacteria 16S rRNA gene were amplified with primers 338F (5′-ACTCCTACGGGAGGCAGCAG-3′) and 806R (5′-GGACTACHVGGGTWTCTAAT-3′) by thermocycler PCR system (GeneAmp 9700, ABI, USA). The Illumina MiSeq platform (Majorbio Bio-pharm Technology Co. Ltd., Shanghai, China) was used. The analysis protocol was performed following our previous work [40]. Sequence analysis was performed by UPARSE softwarepackage using the UPARSE-OTU and UPARSE-OTUref algo-rithms. Sequences with 97% similarities were assigned to the same OTUs. A representative sequence was picked for each OTUand the Greengenes reference database was used to annotate taxonomic information for each representative sequence. In order to compare diversity, the OTU table was rarified, and three metrics were calculated: Chao1, Observed species, and Shannon index. The OTU absolute abundance table was extracted from the pipeline and converted to relative abundances by normalizing to total OTU clustering for analyzing the composition of gut micro-biota and predicting the variation of functional genes by Quantitative Insights into Microbial Ecology software (QIIME) and Phylogenetic Investigation of Communities by Reconstruction of Unobserved States (PICRUSt). A 3D principal coordinate analysis (PCoA) plot was constructed for the evaluation of the dissimilarity and the community composition between samples from unweighted UniFrac distances. Linear discriminant analysis (LDA) effect size (LEfSe) was performed to identify the difference between groups. The correlation analysis between the intestinal bacteria and significant biomarkers in mice was calculated by the R software (R version 3.6.3).

### 4.7. Statistical Analysis

The data obtained from the experiment was analyzed by SPSS Statistics, and the significant difference was declared at *p* < 0.05. The OriginPro 8.0 and GraphPad Prism 6.0 were used for graph analysis. The differences between multiple groups were compared by using Tukey’s test in One-way ANOVA or Two-way ANOVA test for analysis of variance and significance. *p* < 0.05 was considered a significant statistical difference, * *p* < 0.05, ** *p* < 0.01, *** *p* < 0.001, **** *p* < 0.0001.

## 5. Conclusions

Overall, this study strengthens the idea that the MLCT had a faster weight gain effect, compared with the high-fat diet, had no significant difference in lipid metabolism, and had better regulation of glucose homeostasis than the HFD group. This characteristic is different from the traditional MLCT, which provides a reference for the study of the efficacy of MLCT synthesized from lauric acid. At the same time, it is good functionally for people who need to gain weight quickly and replenish energy with a good application prospect in animal husbandry. In addition, the insulin tolerance test and mechanism of anti-inflammatory action can be analyzed in the future in order to elucidate how MLCT can produce anti-inflammatory activity under the condition of reducing insulin resistance. Besides, other types of MLCT can be further synthesized according to the different lengths of carbon chain to explore fatty acids with more functions.

## Data Availability

Not applicable.

## Supplementary Materials

The following supporting information can be downloaded at: https://www.mdpi.com/article/10.3390/molecules28020722/s1, Figure S1: Community barplot analysis at Phylum.; Table S1: The fatty acid composition of MLCT.
