# Supplementary material for: Effects of the Novel LaPLa-Enriched Medium- and Long-Chain Triacylglycerols on Body Weight, Glycolipid Metabolism, and Gut Microbiota Composition in High Fat Diet-Fed C57BL/6J Mice
Source: Molecules. 2023 Jan 11;28(2):722. doi: 10.3390/molecules28020722 (PMC9861698; doi:10.3390/molecules28020722)
Supplement: Supplementary file 1 [file molecules-28-00722-s001.zip › molecules-2089445-supplementary.pdf]

Preparation of MLCT

5 g of algal oil and 8.48 g of LA (molar ratio 1:8) were added into a one-necked round bottom flask (50 mL) capped with a rubber stopper, and incubated at 65 °C in a shaking water bath at 150 rpm. After the substrates mixture were homogenous, TL lipases was added to initiate the reaction as the amount of 8% of total weight of the substrates, respectively. The reaction was held for 3 h, and then the lipases were removed from the substrates by filtration for the collection of products.

Table S1. The fatty acid composition of MLCT

**Fatty acid**	**Contents (%)**	**Sn-2 position contents (%)**
C12:0	50.88 ± 0.18	18.56 ± 0.11
C14:0	0.58 ± 0.03	-
C16:0	34.41 ± 0.45	59.91 ± 0.59
C18:0	2.47 ± 0.05	1.09 ± 0.03
C18:1	9.06 ± 0.22	15.03 ± 0.17
C18:2	2.6 ± 0.07	5.68 ± 0.09

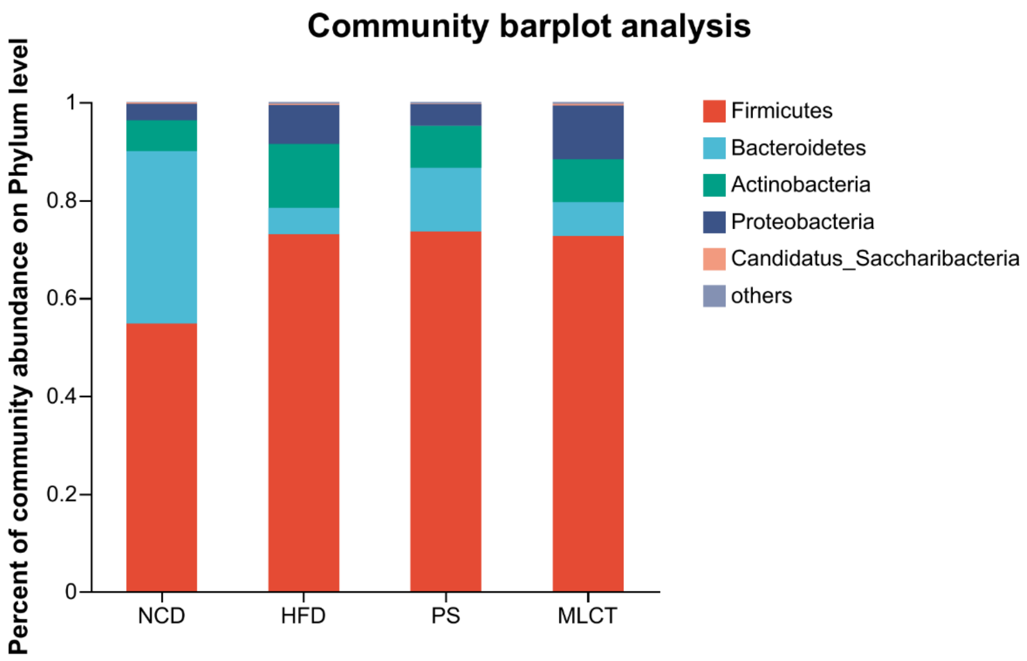

Figure S1. Community barplot analysis at Phylum.
